# Illuminating the Druggable Human Proteome with an AI Protein Profiling Platform

**DOI:** 10.21203/rs.3.rs-7667948/v1

**Published:** 2025-10-03

**Authors:** Guy W. Dayhoff, Daniel Kortzak, Ruibin Liu, Mingzhe Shen, Zhong-Yin Zhang, Jana Shen

**Affiliations:** † Department of Pharmaceutical Sciences, University of Maryland School of Pharmacy, Baltimore, MD 21201, U.S.A.; ‡ Borch Department of Medicinal Chemistry and Molecular Pharmacology, Purdue University, West Lafayette, IN 47907, U.S.A.

## Abstract

Creating a ligandable atlas for the proteome would transform our understanding of protein functions and accelerate therapeutic discovery; however, proteomic approaches are constrained by insufficient proteome coverage and data heterogeneity, while existing machine learning (ML) models have limited power due to structural dependencies and heterogeneous experimental labels. Here we developed AiPP, a multimodal AI platform that predicts and characterizes ligand interaction sites directly from protein sequence. AiPP is powered by the evolutionary-scale protein large language models (LLMs) and leverages two harmonized ML training sets derived from the new databases comprising cysteine ligandability from activity-based protein profiling (ABPP) studies and reversible binding evidenced from co-crystal structures. We developed a LLM representation based clustering framework to interrogate, reconcile, and augment experimental labels in both databases. Two complementary protocols were implemented to iteratively expand the training data while improving model performance. Although trained exclusively on ABPP data, AiPP recovers 80% (Top-1) of cysteine liganding events from cocrystal structures, with 84% AUPRC and 89% AUROC. AiPP recapitulates consistently and heterogeneously liganded cysteines across cancer cell lines and reliably identifies dynamic, ligandable pockets in “undruggable” transcription factors. Remarkably, AiPP accurately predicts active-site and allosteric cysteines in protein tyrosine phosphatases that were undetected by ABPP. Finally, we applied AiPP to the entire human proteome, identifying ligandable sites in proteins that were undetected or unliganded by ABPP, including an allosteric site in MC3R, which is a therapeutic target for treatment of eating disorder and obesity. This proteomewide covalent ligandability atlas (version 1.0) is anticipated to guide future development of chemical probes and pharmaceutical modulators, particularly for understudied proteins and currently undruggable targets. The LLM-based approach to interrogate large-scale heterogeneous data is broadly applicable to protein research and development of proteomics-derived ML models for diverse applications.

## Introduction

The human proteome comprises ~20,300 distinct genes that encode over 200,000 proteoforms^1^ (proteins and their variants arising from splicing, post-translational modifications, single-nucleotide polymorphisms and mutations); however, fewer than 900 proteins have been therapeutically targeted by the FDA-approved drugs (https://go.drugbank.com/).^2^ This gap underscores both the urgent need and vast potential to expand the druggable proteome. First developed as a chemical proteomic approach that utilizes small-molecule covalent probes (known as the activity-based probes) to report on enzyme activities in complex biological systems,^3,4^ activity-based protein profiling (ABPP) has evolved into a powerful and versatile technology. By combining activity- or reactivity-based probes with mass spectrometry (MS) based quantitative proteomics, ABPP has enabled several major advances,^5,6^ including the discovery of reactive cysteines^7^ and characterization of small-molecule–protein interactions (ligandability) in cells,^8^ as well as the development of covalent inhibitors.^9^ In particular, the isotopic tandem orthogonal proteolysis (isoTOP) ABPP experiments utilizing competition ratios (CRs) between covalent probes and fragments first introduced by Cravatt and Weerapana^7,10,11^ have led to quantitative assessment of cysteine ligandability across over 10,000 proteins using different human cell lines^8,12–24^ Recently, the ABPP technology has been further developed for high-throughput screening through minimizing the amount of starting protein materials and instrument time^15^ as well as using label-free quantification.^18,25^

Despite the enormous potential of ABPP and rapid progress made in recent years, several challenges remain, hindering efforts of employing ABPP to create a comprehensive, accurate map of cysteine-directed ligandable sites across the proteome for covalent drug design. One major challenge is limited proteome coverage due to the dependence on protein abundance, probe chemistry, and cell type (e.g., membrane-bound proteins are underrepresented in lysates due to insolubility).^5,25–30^ A second major challenge is the variability in detected proteins, cysteines and their assessed ligandability across the published ABPP datasets (see refs^25,30–32^ and later discussions). This variability may at least in part be attributed to the diverse cellular contexts^25,31^ and to specific experimental conditions,^30^ including probe chemistry and concentration, fragment concentration, treatment duration, cell type (e.g., cell lysates vs. live cells), cellular state (e.g., mitotic vs. asynchronous conditions; protein mutations; post-translational modifications; redox state),^33^ cell line (e.g., different cancers^31^), specific electrophilic fragments used,^25^ and quantification protocols (e.g., MS protocol and CR threshold for defining a liganding event). An additional challenge arises from the inherent limitations of shotgun proteomics,^34^ which prevents the unambiguous identification of a subset of ligandable cysteines.^18,25^ To address these limitations, recent studies implemented label-free and data-independent acquisition protocols,^18,25^ demonstrating improved proteome coverage depth and enhanced data completeness compared to traditional approaches.

In contrast to proteomics approaches which offer broad coverage across the proteome but with significant data variability, X-ray crystallography delivers high-resolution structural information on protein-ligand interactions for a limited number of complexes. Drawing from the cysteine-liganded co-crystal structures deposited in the Protein Data Bank (PDB), several databases (e.g., CovPDB,^35^ CovBinderInPDB,^36^ and LigCys3D^37^) have been developed, which annotate nearly 800 proteins with chemically modified cysteines. Based on these databases and experimental or AlphaFold^38^ structure models, machine learning (ML) models, including support vector models (SVMs),^39^ boosted decision trees,^37,40^ graph neural networks (GNNs),^41^ and convolutional neural networks (CNNs),^37^ have been developed for structure-based prediction of ligandable cysteines, achieving recall and precision as high as 95%. However, these models are not yet deployable for proteome-wide mapping of ligandable cysteine sites due to three critical limitations: (i) the extremely small training dataset and lack of protein diversity; (ii) the absence of reliable controls for unligandable cysteines; and (iii) the requirement for protein structures, which often cannot be met even with state-of-the-art structure prediction tools such as AlphaFold3.^42^

Recently, the ABPP-derived databases (CysDB^43^ and TopCysteineDB^32^) have been developed and used to train structure-based ML models for predicting cysteine reactivity or ligandability.^32,44^ Based on CysDB^43^ and other ABPP data (~800 reactive cysteines) and associated crystal structures, Forli, Backus, and coworkers developed the random forest model CIAA to predict reactive cysteines with an accuracy of 68% in external validation.^44^ Using TopCysteineDB, which combines PDB- (nearly 800 liganded cysteines) with ABPP-derived cysteine ligandability data (over 9,000 ligandable cysteines compiled from three publications^13,15,31^) and experimental or AlphaFold structures, Gohlke and coworkers^32^ developed the XG-Boost model TopCySPAL to predict cysteine ligandability. A unique feature of TopCySPAL is that it is trained with negative cysteines defined as those undetected in liganded proteins quantified by ABPP.^32^ In the 20% hold-out test, TopCySPAL achieved AUROC and AUPRC of 96.4% and 91.4%, respectively. Another structure-based ML model was the CNN developed using the DrugMap database (an ABPP dataset obtained with 412 cancer cell lines and 3 fragments), which achieved AUROC of 69.3% in external evaluation.^31^

Despite recent advances, existing ML models for predicting cysteine ligandability remain severely constrained in both performance and applicability. First, current approaches for developing ML models inadequately leverage ABPP datasets and lack rigorous frameworks for assignment of ligandability labels. These models fail to reconcile the inherent variability in experimental labels, leading to inconsistent training data and unreliable predictions. Second, this reconciliation challenge is compounded by the absence of standardized or rigorous evaluation metrics and benchmarking protocols, making it difficult to rigorously assess model performance or compare different approaches in a meaningful way. Finally, and perhaps most critically, existing models require high-quality protein structures as input, rendering them inapplicable to substantial portions of the proteome that remain structurally unresolved even with state-of-the-art structure prediction tools such as AlphaFold3,^42^ creating a significant coverage gap in ligandability assessment.

To overcome the ML challenges and unlock the full potential of ABPP, we developed a multimodal AI Protein Profiling (AiPP) platform for annotating reversible ligand binding (LigBind) and covalently ligandable residues (LigCys) given a protein sequence. AiPP is powered by state-of-the-art protein large language models (pLLMs), ESM Cambrian (ESMC)^45^ and ESM3,^46^ and two newly curated comprehensive databases, LigCysABPP and LigBind3D. We developed pLLM-based clustering framework to harmonize and augment variable and incomplete experimental data. We further developed iterative learning protocols to systematically expand the ML training set, achieving enhanced model accuracy and generalization. Beyond predicting ligandable sites, AiPP incorporates RIDA,^47^ a disorder annotation platform that identifies disordered molecular recognition features (MoRFs) prone for binding-induced folding. AiPP also incorporates KaML models^48,49^ for predicting cysteine reactivities and solvent accessibilities. AiPP enabled us to extrapolate from the limited experimental data to create the first atlas of cysteine-directed covalent ligandable sites across the human proteome.

## Results and Discussion

### Curation of an ML-ready cysteine ligandability database LigCysABPP.

**Figure 1:**
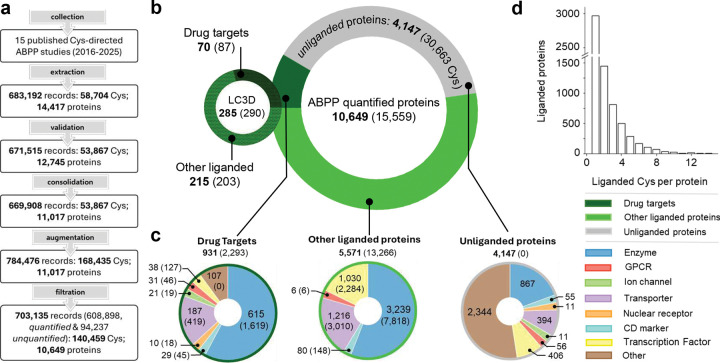
Overview of the ABPP-quantified proteins and cysteines in LigCysABPP database. **a.** The LigCysABPP database is manually curated from a collection of 15 published cysteine-directed ABPP studies.^8,12–25^ Peptide-derived records are extracted from each publication, validated against UniProt, consolidated across identical sequences and subsequences, augmented with unquantified (unseen) cysteine-sites on otherwise quantified proteins, and filtered for compatibility with downstream processing steps. **b.** LigCysABPP contains the cysteine ligandability records of 10,649 ABPP-quantified proteins, of which 6,502 are liganded and 4,147 are unliganded. A liganded cysteine is defined as having at least 1 pos ABPP record while a liganded protein is defined as having at least 1 liganded cysteine. Among the liganded proteins, 931 are known drug targets (with 2,293 liganded cysteines), while the rest of 5,571 other liganded proteins contain 13,266 liganded cysteines. The LC3D database contains 285 unique proteins (290 cysteines liganded in the co-crystal structures), among which only 70 proteins (87 drug targets) are also identified as liganded by ABPP. **c.** Functional classes of drug targets, other liganded proteins, and unliganded proteins according to The Human Protein Atlas classifications^50^(https://www.proteinatlas.org/). **d.** Histogram of the number of ABPP liganded cysteines per protein shows that most liganded proteins contain 1–3 liganded cysteines.

From 15 independent ABPP datasets (also referred to as sources) published between 2016 and 2025,^8,12–25^ we extracted 683,192 peptide-derived records of cysteine ligandability for 58,704 unique cysteine residues across 14,417 proteins in human proteomes. Following data clean-up steps, including protein sequence reconstruction, record validation, and consolidation of UniProt IDs (UIDs) referring to identical protein sequences, the raw entries were converted to 669,908 structured, site-specific cysteine ligandability records for 53,867 cysteine residues across 11,017 proteins. Each record contains the ligandability label (pos for liganded and neg for unliganded) given by the study authors for a specific cysteine site in a protein under a set of experimental conditions and cellular context. To fully characterize the cysteines in ABPP-quantified proteins, we added unquantified (provisionally neg) records for cysteines belonging to the ABPP-quantified proteins but undetected by the probes. To accommodate the ESM-based ML, protein sequences shorter than 30 or longer than 2046 amino acids were removed. This procedure results in 703,135 total curated records describing ligandability (pos, neg, or unquantified) of 140,459 unique cysteine sites across 10,649 distinct proteins (Fig. 1a). Detailed extraction, validation, and consolidation logs are provided in Supplemental Data (SD6–SD11).

### Functional analysis of ABPP-quantified proteins.

We define a liganded cysteine as one with at least one positive ABPP record. A supporting source for a liganded cysteine is one that contains at least one pos record for that cysteine. Among the 10,649 ABPP-quantified proteins, 6,502 are liganded (containing at least one liganded cysteine) and 4,147 are unliganded (Fig. 1b). Among the liganded proteins, 931 (with 2,293 pos cysteines) are known drug targets, while the rest of 5,571 proteins (with 13,266 pos cysteines) represent unexplored potential therapeutic opportunities (Fig. 1b). Among the liganded drug targets and other proteins, enzymes constitute the largest functional family, followed by transporters, nuclear receptors, and others (Fig. 1c). It is noteworthy that transporters and nuclear receptors are substantially more prevalent among other ligandable proteins, each representing over one-third the abundance of enzymes (Fig. 1c). Interestingly, GPCRs are sparsely represented, likely because most cysteine residues are not positioned near ligand-binding pockets. It is intriguing that the majority of unliganded proteins (2,593 out of 4,147) do not belong to any functional class identified by The Human Protein Atlas^50^ (https://www.proteinatlas.org/), suggesting that a significant fraction of the human proteome remains to be functionally characterized.

The overlap between the ABPP-liganded proteins and those evidenced by co-crystal structures in PDB is extremely small, only 60 proteins (42 are known drug targets, Fig. 1b). This limited overlap may be attributed to the historical focus of structural studies on bacterial and other non-human proteins, which are overrepresented in the PDB. The limited overlap also demonstrates the potential of ABPP to greatly expand the known ligandable proteome. The vast ABPP data also allows us to ask a pertinent question: how many ligandable cysteines are in a single protein? Interestingly, while the majority of proteins contain only one ligandable cysteine, a sizable number of proteins harbor 2–4 ligandable cysteines (Fig. 1d), suggesting that cysteine-directed probes or inhibitors may be designed to target allosteric and other ligandable pockets.

### Analysis of ABPP sources and records to assess consensus in cysteine ligandability.

**Figure 2:**
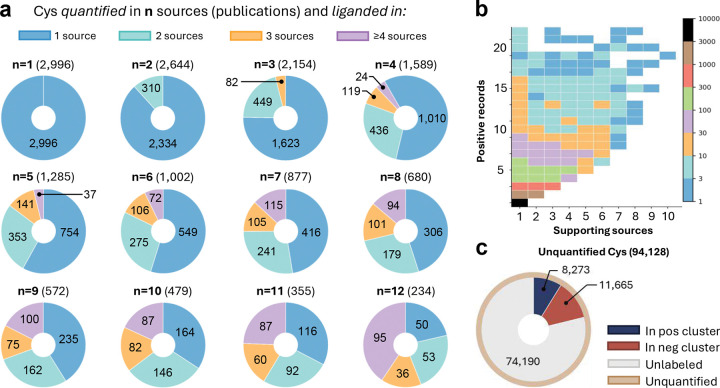
Analysis of cysteine ligandability across ABPP sources and records. **a.** Each pie chart displays the number of liganded cysteines quantified by *n* sources. A liganded cysteine is defined as one that has at least one pos ABPP record. Each pie segment represents the number of cysteines supported by 1 (blue), 2 (green), 3 (yellow), and ≥4 (purple) sources. **b.** The number of liganded cysteines with the number of supporting sources and positive records. A supporting source for a specific cysteine is defined as one that contains at least one pos record for that cysteine. A total of 7,787 cysteines are labeled pos by only 1 record (black). No liganded cysteine is supported by >10 sources. Liganded cysteines with >25 records are negligible and therefore excluded from the plot. **c.** Following representation-based clustering, 8,273 unquantified cysteines are labeled pos, 11,665 labeled neg, while 74,190 cysteines remain unlabeled.

In order to devise an ML labeling scheme based on LigCysABPP records, we first analyzed the consensus among 15 sources for liganded cysteines, which are grouped by the number of sources that quantified them (*n* =1,…,12). For instance, 2,996 cysteines were quantified by a single source, while 2,644 and 2,154 were quantified by 2 and 3 sources, respectively. We then examined the consensus levels by determining how many liganded cysteines are supported by *m* number of sources (*m* =1,…,8). This analysis highlights the challenge of reconciling source-specific labels, as considerable variability exists. For example, among cysteines quantified by 2 sources, only 310 (11%) are supported by both sources, while 2,334 (89%) showed conflicting results – pos in one source and neg in the other. Among cysteines quantified by 3 sources, only 82 (4%) are unanimously supported, while 449 (21%) are pos in 2 sources and 1,623 (75%) are pos in only 1 source. This pattern of decreasing consensus becomes more pronounced as the number of sources increases (Fig. 2a and Supplemental Fig. S1).

In the above analysis, a supporting source can have any number of pos records. To further dissect the ABPP noise, we also examined the number of supporting records for liganded cysteines (Fig. 2b). For liganded cysteines supported by 1 source, 7,787 have only 1 pos record, 1,374 have 2 pos records, and 816 have 3–4 pos records. For liganded cysteines supported by 2 sources, 814 have 3–4 positive records. As the number of supporting sources increases, the number of liganded cysteines with positive records decreases – with 5 sources, the number of liganded cysteines with at least 5 pos records is below 30. The steep decline in well-supported liganded cysteines suggests that requiring agreement among more than 4 sources may be too stringent for practical ML applications, as it would severely limit the training data size.

The significant variation in cysteine ligandability likely reflects differences in experimental conditions (e.g., probe and fragment concentrations) and cellular context (e.g., cell line-specific factors) as shown in SI Data Table S1. The influence of cellular context on cysteine ligandability was recently^31^ demonstrated by an ABPP study of over 400 cancer proteomes, which identified nearly 400 cysteines exhibiting varying degrees of engagement by an identical fragment (“heterogeneous ligandable”). While cellular contexts will be addressed in our future studies, the present work focuses on cysteines that demonstrate consistent labeling patterns across different experiment conditions and cell lines.

### Using representation-based clustering to derive cysteine ligandability labels for ML.

The variability in cysteine ligandability across the ABPP datasets presents a significant challenge for ML. Encouraged by our recent work^49^ demonstrating that residue-level representations (also known as per-token embeddings) from ultra-large pLLMs such as ESMC^45,46^ encode not only evolutionary information but also structural and local biochemical environments, we leveraged the residue-specific representations to reconcile the labels for liganded and unliganded cysteines. Specifically, we performed clustering analysis on all cysteines in ABPP-quantified proteins using pairwise similarity scores computed from ESMC-derived residue-specific embedding vectors. For each cluster, we identified a representative cysteine as the node exhibiting the highest average similarity to all other cluster members. This approach yielded compact, non-overlapping clusters of cysteine sites with high embedding similarity, each anchored by a representative node used for consensus labeling and subsequent model training. To prevent leakage, clusters were never split across data partitions and all clusters sharing a UID were assigned to the same partition.

To derive high-confidence ligandability labels from the ABPP data, we performed cluster-based label assignment on the full set of representation-based clusters according to varying levels of consensus, defined as a combination of supporting sources and records (denoted *n*S–*m*R). For example, under the 4S–4R criteria, a cluster is labeled pos only if it contains at least four positive records from four different sources. Conversely, a cluster is labeled neg if it contains no positive records and at least four negative records from unique sources. Clusters that did not meet either pos or neg labeling condition were excluded from the training set. To augment coverage, unquantified cysteines in quantified proteins cast default negative votes and inherit a cluster-based label.

Applying the 4S–4R criteria yielded 936 pos and 4,590 neg labeled clusters, covering 143,914 site-level records describing 11,869 distinct cysteine residues across 11,541 unique proteins. Importantly, this approach enabled us to assign labels to 21% of ABPP-unquantified cysteines, classifying 8,273 as pos and 11,665 as neg (Fig. 2c). The cluster representatives were then used for downstream model training. Additional details are provided in the Supplemental Methods, including data validation, representation-level deduplication, and procedures with representation-based criteria to prevent data leakage between training, validation, and external evaluation.

Note, while conventional sequence identity filtering methods, such as CD-HIT,^51^ focus on eliminating sequence similarity, they fail to account for functionally convergent or structurally similar ligandable sites that may exist within otherwise divergent proteins. In contrast, clustering based on pLLM representation goes beyond simple sequence comparison by integrating local biochemical, structural, and evolutionary information. Thus, representation-based criteria are more stringent and effective in reducing the risk of data leakage.

### Support from 4 sources may be an optimal tradeoff between label confidence and data coverage.

**Figure 3:**
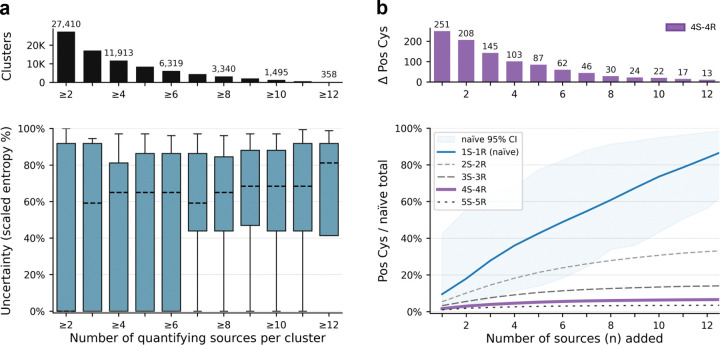
Analysis of how source threshold affects label variability and data coverage. **a.** *Top:* Bar plot showing the number of cysteine clusters supported by at least *n* independent sources, regardless of the number of underlying records. For clarity, bins corresponding to odd values of *n* are unlabeled. *Bottom:* Label variability within each cluster, stratified by the number of supporting sources. Variability is quantified using the Shannon entropy of the binary label distribution (*p*, the fraction of sources labeling a cysteine as positive), scaled to a 0–100% range. Boxes denote the interquartile range; medians are shown as horizontal dashed lines; whiskers span the 10th–90th percentiles. A minimum in entropy is observed at *n* ≥ 4, suggesting this threshold maximizes labeling consistency across sources. **b.** *Top:* Incremental gain in the number of positive cysteines when increasing from *n*–1 to *n* sources. *Bottom:* For each consensus threshold (e.g., 1S–1R through 5S–5R), we randomly sample *n* sources from a pool of 15, repeat 200 times, and compute the average number of positive cysteines. This is normalized by the naïve total (i.e., all cysteines reported as positive in any single record). The 95% confidence interval is shown for the naïve curve. The 4S–4R consensus curve (highlighted in purple) plateaus after four sources, supporting its use as a data-driven threshold for robust labeling.

To examine how label confidence depends on the number of quantifying sources, we measured variability using Shannon entropy (a measure of labeling uncertainty) of the binary pos/neg distribution for cysteine clusters quantified by *n* sources. The number of clusters falls sharply as the threshold increases; for example, requiring at least 4 quantifying sources reduces the set to 11,913 clusters compared to 27,410 with a 2-source threshold (Fig. 3a, top). Entropy is high for clusters supported by 2 or 3 sources, reaches its minimum at 4 sources (Fig. 3a, bottom), and then rises again at thresholds ≥ 7, peaking at 12 sources where only ~150 highly heterogeneous clusters remain.

Fig. 3b complements this analysis by showing how positive cysteines accumulate as additional sources are added under different consensus rules (1S–1R, 2S–2R, …, 5S–5R). At the 4-source threshold, the number of positives normalized by the naïve total (all cysteines reported as liganded in any record) qualitatively plateaus: each additional source contributes relatively few new positives (Fig. 3b, top). Together, these results suggest that requiring evidence from at least 4 supporting sources (defined here as quantifying sources that reported a cysteine as liganded at least once; the 4S–4R rule) provides a practical balance between label confidence and data coverage for annotating ligandable cysteines with the currently available data.

### Development of the sequence-only LigCys models.

Inspired by our recent demonstration that ESMC-based, sequence-only models can accurately predict residue-specific p*K*_*a*_ values, particularly for cysteines despite limited training data,^49^ we developed the LigCys model for predicting cysteine ligandability. The model employs ESMC as the foundational pLLM, using frozen 2,560-dimensional per-residue embeddings passed through a three-layer multilayer perceptron (MLP) classifier trained on the ABPP-derived data described below. Embeddings from layer 76 of ESMC were used (Methods), as preliminary testing showed this layer provided optimal performance for LigCys predictions. Consistent with prior observations,^49,52^ different transformer layers capture distinct evolutionary and structural features at different rates; layer 76 embeddings were therefore also used for LigBind models. We also evaluated pLLM fine-tuning approaches using ESM2,^53^ but the resulting models did not outperform the representation-only ESMC approach for cysteine ligandability prediction.

### LigCys models trained on the 4S–4R ABPP dataset show the strongest generalization.

Due to the highly variable nature of ABPP data, both model training and validation are extremely challenging. To address validation challenges, we used crystallography-validated ligandable cysteines from the LC3D database as an external test set. LC3D cysteines and any representation-based clusters containing these cysteines were removed from the training set to prevent data leakage (Supplemental Methods). Therefore, the LC3D test set is not only orthogonal to the ABPP training set but also provides the most stringent validation for model’s ability to generalize.

We evaluated how ABPP data consensus requirement affects the model performance by training LigCys models with ligandability labels derived from increasingly stringent consensus criteria (Table 1). These LigCys models are compared using the standard metrics, including the most pertinent metric Top-1 recovery, which is defined as the probability that the cysteine with the highest predicted classification score in a protein is a true pos given that the protein has at least one pos cysteine. Since LC3D negatives reflect the absence of observed liganding event rather than validated inactivity, AUROC/AUPRC should be interpreted with caution; we therefore emphasize ranked recovery metrics (e.g., Top-1). Confirming the substantial heterogeneity across ABPP data, the 1S–1R model, despite being trained on the largest dataset (13,430 pos and 55,441 neg cysteines), delivers the worst performance, with AUROC, AUPRC, and Top-1 recovery values of 72.2%, 60.0%, and 40.5%, respectively. At the single-source level, performance improves dramatically as the number of required pos records increases, reaching AUROC, AUPRC, and Top-1 recovery of 87.3%, 81.2%, and 71.7%, respectively, despite a ten-fold reduction in training data. A similar trend of significant performance gain with increasing number of pos records is observed at the 2- and 3-source levels. However, at the 4-source level, performance with 5 pos records (4S–5R) is very similar to that with 4 records (4S–4R), which has the AUROC, AUPRC, and Top-1 of 87.3%, 81.2%, and 75.2%, suggesting that model performance may be plateaued. Indeed, the 5-source model (5S–5R) shows decreased performance across all three metrics by 2–5% compared to the 4S–5R model, which may be attributed to the substantial (more than 50%) reduction in training data (only 142 pos and 236 neg cysteines).

This comparison shows that the 4S–4R training set yields the best model performance (Table 1, highlighted row) for generalization to the completely orthogonal external validation data. This observation is consistent with the analyses in Fig. 2 and 3, suggesting that the 4S–4R training set balances label confidence with dataset size for effective generalization. To further assess model performance, we computed Top-1* recovery for the LC3D test set that received additional label assignments through representation-based clustering among themselves (referred to augmented LC3D or LC3D*). The 4S–4R model remains the top-performing one, achieving AUROC, AUPRC, and Top-1* of 87.3%, 81.2%, and 79.4%, respectively.

### Enhanced model performance through iterative training data expansion guided by LC3D-based evaluation.

Although the 4S–4R dataset achieves superior performance relative to others, its small size may limit the model’s generalization capability. To address this limitation, we hypothesized that using LC3D as a validation benchmark would allow us to identify additional cysteines (and proteins) with high-confidence labels from the broader ABPP dataset to expand the training data. To test this hypothesis, we developed an iterative procedure guided by LC3D Top-1 recovery, starting from a truncated version of the high-confidence 4S–4R dataset containing cluster representatives from proteins with both positive and negative cysteines. In this truncated version (denoted 4S–4R^*t*^), 23 proteins quantified by 10 or more sources with at least one pos and one neg cysteine are withheld for model evaluations. The candidate training pool included cysteines from all 4S–4R^*t*^ clusters, extending beyond the baseline to encompass non-representative members as well as proteins supported exclusively by positive or exclusively by negative evidence.

In iterations 1–3, we generated 100 batches, each consisting of 80, 100, and 150 randomly selected cysteines from the candidate pool, respectively. From iteration 4 onward, we generated 100 batches of 175 randomly selected cysteines. In each iteration, batches were evaluated by incorporating them into the training set and training ensembles of 24 models (6 data splits with 4 random seeds per split). The best-performing batch was selected for further validation using 10 new random splits with 20 models per split, with Top-1 recovery serving as the evaluation metric. If Top-1 recovery exceeded the previous iteration’s baseline, the best batch was accepted.

Through six iterations, Top-1 recovery increased from 70.9% to 79.9% on LC3D and from 75.1% to 84.5% on LC3D*, accompanied by steady improvements in AUROC from 84.5% to 88.6% and AUPRC from 77.8% to 84.2% (Table 2). In iteration 7, no further gain was observed, indicating that performance had plateaued under this random-batch expansion scheme. Hereafter we will call the Iter6 model LigCys-S. Relative to the baseline 4S–4R^*t*^ dataset, the expanded training set nearly tripled in size to 1,099 proteins, driven by over twofold increase in negative cysteines (1,345) and a nearly 30% increase in positives (441). Using the same data, we trained a structure-aware model (6-SA) to test if adding additional structural features would enhance predictive power (Table 2, row 6-SA). Interestingly, along with other calculated metrics, the Top-1 recovery of LC3D liganded cysteines is decreased by 1.7 %. This suggests that the evolutionary driven pLLM-based representations capture the majority of information pertinent to cysteine ligandability.

### Enhanced model performance through iterative training data expansion guided by ABPP-based evaluation.

As random sampling in the LC3D-guided procedure may leave parts of the candidate pool unexplored, we hypothesized that a systematic, cross-validation (CV) driven approach could more efficiently identify additional high-confidence data. To test this hypothesis, we developed an expansion strategy guided by CV performance on the ABPP data, starting from the same baseline dataset (4S–4R^*t*^) as in the LC3D-guided expansion. The same candidate data pool was also used.

At each iteration, candidate batches of fixed composition (25 positives and the remainder negatives) were assembled, with batch size set to 100 cysteines in early iterations and increased to 175 as the pool expanded. Batches were prioritized by ensemble uncertainty, and a batch was admitted only if it significantly exceeded the AUPRC enrichment (PRCE) of the previous iteration baseline in the CV. PRCE (enrichment of AUPRC over random guess) was used due to the variable pos:neg Cys ratio in each iteration. To broaden the search, advancing batches were further refined by simple genetic algorithm steps, preserving the best performers while generating crossover and mutation variants.

Expansion proceeded through four tournaments, each comprising four iterations. Here, a “tournament” refers to a series of evaluations in which candidate batches compete against the baseline: top performers are retained and recombined, and only the strongest batch is accepted at each iteration of the tournament. Expansion terminated when the final tournament ended without any accepted batch. This acceptance scheme yielded steady performance gains: Top-1 recovery stayed constant at 70.9% on LC3D and at 75.1% to 76.4% on LC3D*, with corresponding increases in AUROC and AUPRC (Table 2, under ABPP-guided expansion). Hereafter we will refer to the Tour4 model as LigCys-A. The final expanded set comprised 1,744 proteins and 2,633 cysteine residues, representing a 1.8-fold increase in positives and a 3.4-fold increase in negatives relative to the 4S–4R^*t*^ baseline. Performance gains plateaued after the fourth tournament, indicating that the available candidate pool had been exhausted. As in the LC3D-guided expansion, we also trained a structure-aware model using the final expanded data. The resulting model (4-SA) has a small increase of 0.7% for AUPRC and 0.4% Top-1 recovery compared to the sequence-only model.

Beyond the LC3D test, we evaluated the LigCys-S and LigCys-A models against the ABPP hold-out set comprised of 23 proteins quantified by 10 or more sources, each containing at least one positive and one negative cysteine. From this hold-out set, we created 9 test sets using the *n*S-*n*R consensus thresholds (*n*=1,…,9). As expected, the number of labeled cysteines decreases from 1S-1R (~300 cysteines) to just under 100 at 4S-4R and 7 at 9S-9R (Supplemental Fig. S2). The AUPRC of both models increases with the consensus level of the test set, from ~65% (66 for LigCys-A model) at 1S-1R to 73% (71 for LigCys-A model) at 4S-4R and nearly 100% at 9S-9R (Supplemental Fig. S2). Similarly, the model recall steadily increases from below 40% at 1S-1R to 100% at 9S-9R (Supplemental Fig. S2). These improvements stem from increased TPs and decreased FNs with growing consensus level (Supplemental Fig. S2). The analysis demonstrates that the models perform better on consistently liganded cysteines, corroborating our initial findings (Table 1). Consistent with the LC3D test, the ABPP hold-out test suggests that LigCys-S and LigCys-A models perform similarly, with LigCys-S showing slightly fewer false positives when predicting highly ligandable cysteines. Hereafter, LigCys refers to the blended model.

### Development of an ML-ready LigBind3D database and the sequence-only LigBind models for predicting reversible ligand binding residues.

Since truly ligandable cysteines are located at or near a reversible binding pocket, we developed sequence-only LigBind models (LigBind-seq) to predict reversible ligand-binding residues complementary to cysteine ligandability predictions. To develop the LigBind models, we created an ML-ready database called LigBind3D based on BioLiP2,^54^ an updated manually curated dataset of experimental structures from biologically relevant protein–ligand complexes in the PDB. BioLiP2^54^ is significantly larger than commonly used datasets for protein-ligand affinity predictions (e.g., BindingDB^55^) which contain only protein–ligand interactions with experimentally determined binding affinities.

We selected BioLiP2 entries of monomeric proteins containing small molecules with molecular weights between 150–600 Da and applied a custom structural annotation pipeline (PickPocket, unpublished) to validate biologically relevant protein-ligand interactions. Following the various filtering steps, we reconstructed the full protein sequence directly from the atomic coordinate file. Ligand-binding residues were labeled based on a 4.5 Å heavy-atom distance cutoff. These residues were converted into structured, site-level records. Details are given in Supplemental Methods. The final LigBind3D database comprises 687,712 site-level records across 1,998 unique proteins. This database offers a direct structural perspective on reversible protein-ligand interactions, complementing the LigCysABPP and LC3D databases. In contrast to LigCys3D, which restricts to cysteines, LigBind3D labels every residue in a protein, reflecting the broader diversity of reversible protein-ligand interactions. Similar to the treatment of LigCysABPP data, representation-based clustering was applied to the LigBind3D data for creating the training, validation, and test datasets.

The LigBind-seq model employs a simpler single-layer feedforward network architecture that processes fixed 2,560-dimensional pertoken representations extracted from ESMC layer 76. Preliminary screening showed that the simpler architecture was sufficient to capture ligand-binding patterns from sequence alone. In addition to the sequence-only models, we also developed structure-aware LigBind models (LigBind-SA) by incorporating 3D geometric features through a pretrained transformer model derived from the PeSTo architecture^56^ alongside the pLLM embeddings. Details are given in Supplemental Methods.

In the hold-out test, LigBind-seq achieves AUROC, AUPRC, and Top-10 scores of 93.9%, 74.5%, and 78.1%, respectively, while LigBind-SA attains 95.1%, 77.5%, and 80.1% (Table 3). The modest performance improvements of LigBind-SA relative to LigBind-seq indicate that the ESMC-derived representations provide the majority of the model’s predictive power.

### LigBind predictions offer complementary validation for LigCys predictions.

**Figure 4:**
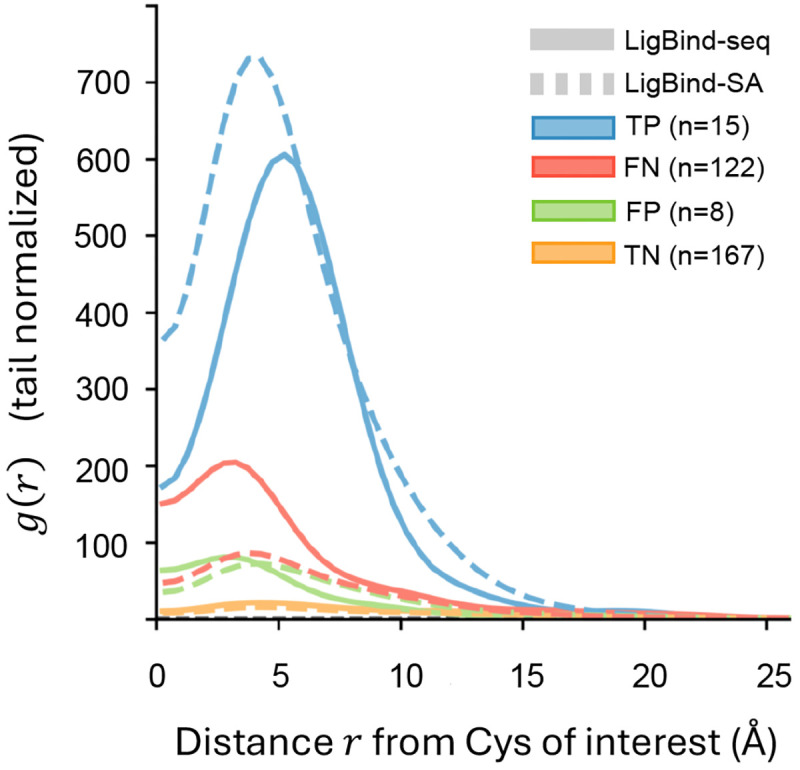
Radial distribution function of LigBind-predicted ligand-binding residues around cysteine sites, stratified by the LigCys prediction outcome (TP, TN, FP, FN). Distance is the minimum all-atom Euclidean distance between the cysteine of interest and each predicted ligand-binding residue. Radial distribution function *g*(*r*) is normalized by the mean shell density over the last 20% of the distance range. Solid and dashed lines represent the LigBind-seq and LigBind-SA predictions, respectively, on the pruned LC3D test set that excludes proteins in the LigBind training set.

Covalent ligandability of cysteines requires proximity to a reversible binding pocket. Therefore, accurate LigBind predictions not only provide reversible pocket information but may also serve as complementary validation for LigCys predictions. To test this complementary relationship, we applied both LigBind and LigCys models to the LC3D test set with proteins in the LigBind training set excluded. We then calculated the radial distribution function (RDF) of predicted ligand-binding residues surrounding TP, TN, FP, and FN cysteines (Fig. 4). Both LigBind-seq and LigBind-SA models were used. Except for TNs, all RDFs exhibit a peak around 4–6 Å. Notably, both LigBind-seq and LigBind-SA RDFs for TPs show the highest peak, consistent with the expectation that ligandable cysteines are proximal to reversible binding pockets. The peak of the LigBind-SA RDF (dashed) is a bit higher compared to the LigBind-seq RDF (solid), suggesting that incorporating structural features enhances pocket prominence. Reassuringly, both LigBind-seq and LigBind-SA RDFs for TNs lack peaks, consistent with the expectation that unligandable cysteines are not located near ligand-binding pockets. Remarkably, the LigBind-seq RDF for FNs exhibits the second highest peak, suggesting that LigBind-seq predictions could help identify overlooked ligandable cysteines based on pocket proximity. Conversely, the LigBind-seq and LigBind-SA RDFs for FPs display very low peaks, suggesting that incorrectly predicted ligandable cysteines can be filtered out based on their lack of proximal binding pockets. These results demonstrate that integrating LigCys and LigBind predictions within a multimodal framework would further enhance the reliability of covalent ligandability assessment.

### Development of the AiPP platform for sequence-based predictions of reversible and covalent ligand binding sites across the proteome.

**Figure 5:**
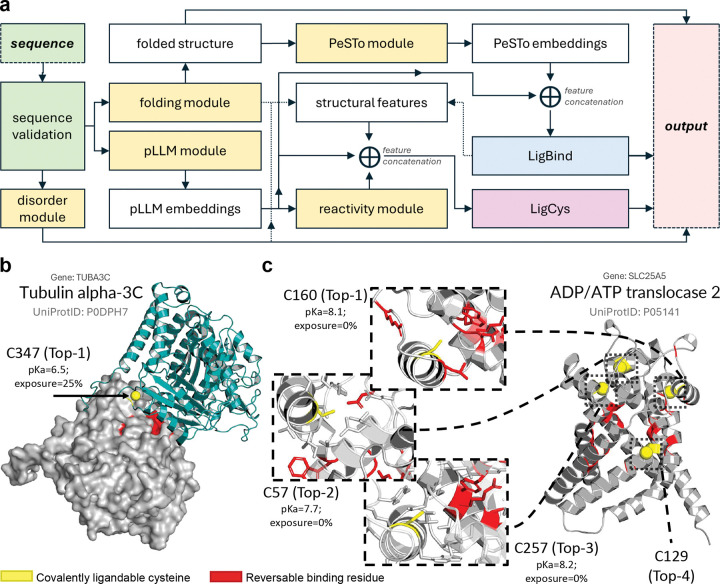
Architecture of AiPP and recapitulation of consistently and heterogeneously liganded cysteines across cancer cell lines. **a.** Architecture of AiPP. Starting from a protein sequence, AiPP generates a predicted 3D structure (ESM3), structural and functional disorder annotations (RIDA), and per token ESMC representations. The latter are processed by three modules: LigCys (cysteine ligandability), LigBind (reversible binding residues), and KaML-ESM (cysteine reactivity), and results are mapped onto the ESM3-predicted structure. Optionally, the predicted structure is processed by the PeSTo module to generate geometric representations, which are then integrated with the ESMC-based representations as well as hand-engineered features to generate LigCys-SA and LigBind-SA predictions. **b.** Prediction for TUBA3C, which contains a consistently liganded cysteine (C347, Top-1 ligandable by LigCys) according to an ABPP experiment across cancer cell lines.^31^ Top-20 reversible binding residues predicted by LigBind are colored red. C347 is located at the binding interface with *β*-tubulin (gray surface) from the adjacent *α/β*-tubulin dimer, the fundamental repeating unit of microtubule protofilaments. The structure of *β*-tubulin is adopted from the electron diffraction model (PDB ID: 1TUB).^57^ **c.** Prediction for SLC25A5, which was suggested to contain heterogeneously liganded cysteines across cancer cell lines.^31^ LigCys predicted C57, C160, C257, and C129 as Top-1, Top-2, and Top-3 ligandable, respectively, consistent with the fragment hits.^31^ The ligandable cysteines are further characterized by the predicted p*K*_*a*_ and % solvent exposure.

Building on the LigCys and LigBind models, we developed the Artificial Intelligence Protein Profiling (AiPP) platform as a unified, sequence-based framework for proteome-wide ligandable site identification (Fig. 5). AiPP integrates reversible and covalent ligand-binding predictions as well as a disorder predictor to support rational drug discovery. The LigBind module detects residues that spatially stabilize ligand binding; the LigCys module identifies ligandable cysteines and ranks them based on prediction scores; and the RIDA module (based on the RIDAO server^47^) predicts per-residue disorder and molecular recognition features (MoRFs) that can fold upon binding. Together, these predictions map ligandable binding sites guiding cysteine-directed covalent ligand design, while simultaneously identifying dynamic or induced-fit binding regions amenable to PPI inhibitor development. Additionally, the recently developed KaML-ESM^49^ and KaML-CBtree^48^ models are incorporated to offer reactivity information for cysteines of interest based on sequence only or structure.

Starting from a protein sequence, AiPP generates a predicted 3D structure (via a local version of ESM3^46^), a structural and functional disorder annotation (via a local version of RIDAO server^47^), and the residue-specific representations (from the pLLM ESMC^45^). The ESMC-based representations are processed by LigBind and LigCys modules to generate predictions of reversible and cysteine-directed covalent ligand binding sites, respectively. LigCys generates predictions from the LigCys-S and LigCys-A models, as well as a blended prediction that ensembles results from both models. Results are visualized on the ESM3 predicted structure with potential MoRFs. Optionally, the predicted structure is processed by a PeSTo^56^ derived transformer to generate geometric representations, which are integrated with the ESMC-based representations as well as hand-engineered features to output LigBind-SA and LigCys-SA predictions. Additionally, cysteine reactivity (using KaML-ESM predicted p*K*_*a*_ as a proxy) and solvent accessibility (calculated based on predicted structure) are annotated to inform ligand design.

While our evaluation of LigCys models (Table 2) emphasized Top-1 recovery, real-world applications would benefit from all predicted positive cysteines. Below we illustrate the practical utility of AiPP by discussing several use cases.

### Application case study 1: AiPP corroborates consistent and heterogeneous ligandability patterns across cancer cell lines.

We applied AiPP to examine 6 proteins that are not in the training set and highlighted in the DrugMap publication (Figure 2)^31^ as containing cysteines either consistently or heterogeneously labeled across cancer cell lines (Supplemental Data file 2). LigCys predicted TUBA3C C347 as Top-1 ligandable with a high score (Fig. 5b), corroborating the consistent engagement across cancer cell lines.^31^ Cys347 is positioned at the binding interface with *β*-tubulin from the adjacent dimer^57^ in the protofilaments that comprise the microtubule structures. The convergence of AiPP and ABPP evidence^31^ for Cys347 ligandability suggests an opportunity for disrupting or stabilizing this protein-protein interaction (PPI). LigCys predicted ACAT1 C126 as the Top-1 highly ligandable cysteine followed by C413 and C193; these cysteines have similar number of fragment hits according to the DrugMap web server (https://drugmap.net).

While the consistently labeled cysteines were always predicted as positives, the heterogeneously labeled ones^31^ were predicted as positives or negatives (Supplemental Data file 2). SLC25A5 (C257) and PCBP1 (C109) were each predicted as Top-3, whereas LARS1 (C70), and RPS3A (C201) were predicted as negative. Notably, the prediction scores of the top 4 cysteines in SLC25A5 (C160, C57, C257, and C129) support the ranking based on whether the cysteine engages all three fragments and total number of hits (Fig. 5c). In particular, both model and ABPP identify C160 as highly ligandable and C129 as very weakly ligandable. Although our model is agnostic to cell lines, these data show that predicted strong and weak ligandabilities correlate with consistent and heterogeneous ligandability patterns observed across cell lines, respectively.

### Application case study 2: AiPP identifies and prioritizes covalent ligandable sites in transcription factors (TFs).

TFs are traditionally considered “undruggable” due to the lack of well-defined pockets and large, disordered regions that bind DNA or other proteins. AiPP is particularly valuable for TFs, as it integrates ligandable cysteine predictions with pocket and MoRF evaluations. To illustrate this utility, we applied AiPP to the TFs highlighted in the DrugMap publication (Figure 5)^31^ but not in the training.

The LigCys predictions are consistent with the total fragment hits reported by the DrugMap server (https://drugmap.net)^31^ (Supplemental Data file 2). LigCys identified FOXA2 C247, SOX10 C71, and TFAP2C C209 as Top-1 or near Top-1 cysteines, consistent with the largest number of hits for each protein.^31^ LigCys identified MYB C374 and IKZF1 C254 as Top-2 cysteines and IRF4 C194 as a Top-5 cysteine, consistent with the very small number of hits for each protein. Interestingly, the predicted Top-1 cysteine in PAX8 (C45) is ranked second based on hits, while C57, which has the highest number of hits, was ranked Top-3 by LigCys.

Among the 8 TFs, the only (seeming) discrepancy is MYOD1 C135, which was predicted to be unligandable. This prediction is however consistent with the extremely low number of fragment hits, 2 out of 1,236.^31^ Intriguingly, LigCys identified an alternative site, C251, as the Top-1, highly ligandable cysteine in MYOD1, despite it not being detected in any ABPP studies. Notably, AiPP predicted C251 to lie in a MoRF region, suggesting potential binding-induced folding. MYOD1 is known to regulate the expression and circadian amplitude of the core clock component Bmal1.^58^ Therefore, C251 may be a candidate site for covalent ligand discovery targeting MYOD1.

**Figure 6:**
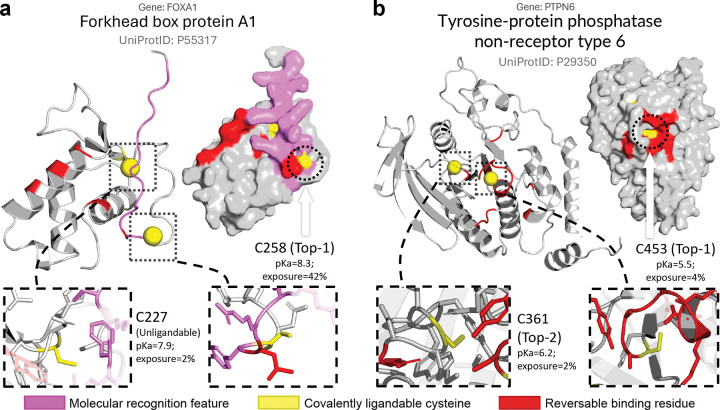
AiPP reveals ligandable cysteines and associated dynamic pockets in undruggable transcription factors and protein tyrosine phosphatases. **a.** The Top-1 ligandable cysteine (C258) in FOXA1 was recently labeled by a stereo-selective acrylamide probe, leading to changes in chromatin binding.^59^ C258 is located in the predicted MoRF region (magenta), which contains a reversible binding residue (red). In contrast, C227 was predicted as unligandable and lacks proximity to MoRF regions or reversible binding residues (negative control). **b.** LigCys identified the active-site (C453, positive control) and allosteric (C361) cysteines in PTPN6 as Top-1 and Top-2 ligandable, respectively. In contrast, PTPN6 C453 is undetectable by ABPP (DrugMap).^31^ LigBind mapped reversible binding residues (red) near these cysteines, providing guidance for ligand design. The Top-5 and Top-20 reversible binding residues are shown for FOXA1 and PTPN6, respectively.

FOXA1 and FOXA2 are two related pioneer TFs that play critical roles in various cancers. LigCys identified C257 in FOXA1 (and analogous C247 in FOXA2) as the Top-1, highly ligandable site. This cysteine was also predicted (by RIDA) to be next to a flexible, unstructured MoRF region and surrounded by a (LigBind) predicted reversible ligand-binding residue, suggesting that its ligandability is enabled by a dynamic pocket (Fig. 6a). Indeed, an acrylamide-based stereoselective probe has been recently discovered that labels FOXA1 C257 in a DNA dependent, remodeling its activity in prostate cancer cells.^59^

Interestingly, a second cysteine in FOXA2, C216 (corresponding to FOXA1 C227), was highlighted as ligandable in the DrugMap publication,^31^ while this cysteine in both FOXA1 and in FOXA2 was predicted as negative and not near a MoRF region or reversible binding residue (Fig. 6a, Supplemental Data file 2). Consequently, AiPP would deprioritize this second cysteine in ligand discovery campaigns, which is corroborated by the experimental data in Ref.^59^ The successful application of AiPP to TFs establishes its utility for guiding ligand discovery against undruggable targets that lack well-defined pockets.

### Application case study 3: AiPP reliably identifies active-site and allosteric cysteines in protein tyrosine phosphatases (PTPs) undetectable by ABPP.

Despite being key cell signaling regulators implicated in several cancers, PTPs have long been deemed undruggable.^60^ PTPs utilize a conserved cysteine (e.g., C215 in PTPN1, C453 in PTPN6, and C459 in PTPN11) for catalysis. Unsurprisingly, this active-site cysteine has been targeted by several covalent inhibitors, including a seleninate, which labels C215 in PTPN1.^61^ Remarkably, LigCys recapitulated the active-site cysteine as Top-1 ligandable in PTPN1 and PTPN6 and as Top-2 ligandable with a similar score as Top-1 cysteine (C318) in PTPN11 (Fig. 6b, Supplemental Data file 2). In contrast, data from DrugMap (https://drugmap.net) indicate that PTPN1 C215 shows very weak ligandability, while PTPN6 C453 and PTPN11 C459 are undetectable by the activity-based probe.

LigCys also identified a second conserved cysteine (PTPN1 C121, PTPN6 C361, and PTPN11 C367) in a disordered loop as Top-2 (PTPN1 C121 and PTPN6 C361) or weakly (PTPN11 C367) ligandable (Fig. 6b, Supplemental Data file 2). Interestingly, while PTPN1 C121 was also ranked as the second ligandable cysteine according to DrugMap, PTPN6 C361 is barely ligandable and PTPN11 C367 was not detected. LigBind revealed that the active-site cysteine is not only surrounded by numerous reversible binding residues but also itself reversible binding, while the Top-2 cysteine is located near a reversible binding residue (Fig. 6b). Covalent modification of C121 in PTPN1 has been shown to inactivate the enzyme,^62^ suggesting that the cysteine is located near an allosteric pocket, which supports the LigBind prediction. Interestingly, we have discovered a propynone-based covalent inhibitor for PTPN6, which targets the corresponding C361, further supporting the existence of an allosteric pocket in PTPs (unpublished data). The prospective identification of both active-site (positive control) and allosteric cysteines in PTPs further validates the practical utility of AiPP for guiding ligand discovery. The fact that most of these cysteines were undetected or missed by ABPP demonstrates a key advantage of the AiPP platform.

### AiPP illuminates ligandable sites across the human proteome, including drug targets undetected or unliganded by ABPP.

**Figure 7:**
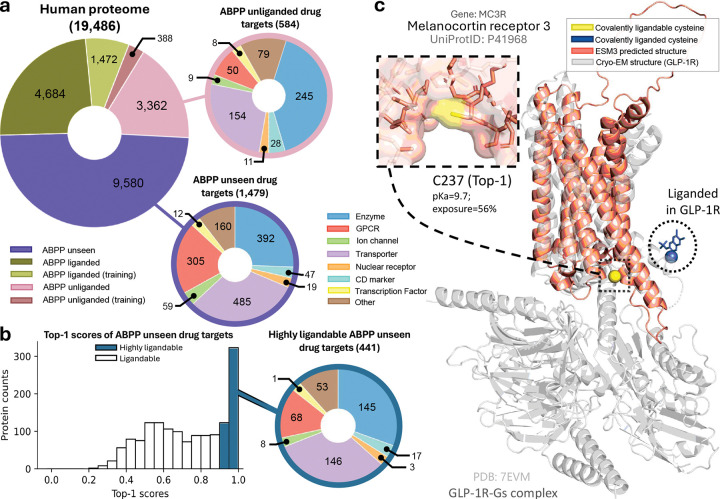
AiPP illuminates ligandable sites across the human proteome, including drug targets undetected or unliganded by ABPP. **a.** Of the 19,486 unique proteins in the human proteome (UniProt database https://www.uniprot.org/), 9,580 have not been detected and 3,362 have not been liganded by ABPP (as in the LigCysABPP database). A total of 1860 proteins (1472 ABPP liganded and 388 ABPP unliganded) are in the model training set. Drug targets within ABPP undetected or unliganded proteins are stratified by functional family classifications. **b.** A histogram of Top-1 cysteine ligandability scores for drug targets undetected by ABPP. The pie chart displays functional families for those predicted to contain a highly ligandable cysteine (Top-1 score > 0.90). The proteomewide AiPP ligandability data are accessible at https://aipp.computchem.org. **c.** AiPP predicts a highly ligandable cysteine (C237^6.30^) in MC3R (predicted structure in green). In support of this allosteric pocket, an analogous cysteine (C347^6.31^) in GLP-1R was labeled by a sulfonamide compound.^63^ Supplemental Fig. 3 and 4 provide more details.

Having extensively validated the extrapolation power of AiPP, we applied it to the entire human proteome (Fig. 7a). AiPP assessment covers all 19,486 unique proteins in the human proteome (UniProt database), which is more than ten fold of the LigCys training set (1,472 ABPP liganded and 388 ABPP unliganded proteins) and includes 9,580 APP undetected and 3,362 APP unliganded proteins (as in the LigCysABPP database). AiPP illuminates drug targets that were undetectable by ABPP, including 305 GPCRs, 485 transporters, and 392 enzymes (Fig. 7a). Notably, 441 of the undetected drug targets are highly ligandable (LigCys Top-1 score > 0.9), including 68 GPCR targets (Fig. 7b).

One emerging GPCR drug target is melanocortin receptor 3 (MC3R), which is expressed in multiple brain regions and regulates feeding behavior and adiposity.^64,65^ LigCys predicted a highly ligandable cysteine, C237^6.30^, positioned at the cytoplasmic terminus of transmembrane helix 6 (TM6). A highly ligandable cysteine was predicted at a similar position in NMUR2 (C232^6.29^), which is also involved in appetite regulation.^66^ Intriguingly, although not predicted by LigCys, the analogous C347^6.31^ in GLP-1R was labeled by a sulfonamide compound, leading to allosteric activation or positive modulation of the receptor.^63^ Overlay of the ESM3-predicted structure of MC3R (inactive state) with the cryo-EM structure of GLP-1R/Gs complex suggests that a liganding event at C237^6.30^ directly impact G-protein coupling (Fig. 7c). In support of this allosteric location, LigBind identified nearby reversible binding residues for GLP-1R, MC3R, and NMUR2. Collectively, the AI predictions and experiment data support the existence of an allosteric pocket in MC3R and NMUR2, offering a testable hypothesis for development of covalent as well as reversible small molecules to modulate their functions.

## Concluding Discussion

AiPP is the first multimodal AI platform for accurate, sequence-based, scalable annotation of reversible and covalent ligand-binding sites. This platform enabled us to provide the first comprehensive atlas of cysteine-directed covalent ligandable sites across the human proteome. AiPP is powered by state-of-the-art pLLMs, new ML-ready databases, and methodological advances that address and overcome the critical challenges in proteome-wide ML based on heterogeneous experimental data.

We addressed a critical data challenge through a representation-based clustering framework to reconcile and augment experimental labels and a rigorous consensus analysis approach to distill a baseline training set. We implemented two iterative approaches to systematically increase training data, achieving substantial improvements in model performance and generalization. We eliminated structure requirement by leveraging state-of-the-art ESMC^45^ as the foundation model. Importantly, AiPP integrates multiple complementary modalities to provide comprehensive profiling of cysteine sites, which can serve as targets for both covalent and reversible ligand design.

AiPP can be systematically improved in the future, e.g., by incorporating continuously growing data from ABPP experiments and by adding capabilities for predicting covalent ligandability of lysine and other residue sites. Although limited comparison to the recent DrugMap work^31^ suggest that LigCys models may already capture some information about heterogeneous ligandability through the prediction ranking system, current LigCys models are currently agnostic to cellular context, e.g., redox conditions, post-translational modifications, and mutational states. Two recent ABPP studies revealed that phosphorylation^33^ and specific cancer-cell environment^31^ can significantly modulate cysteine reactivity and/or ligandability in a subset of cysteines. The underlying mechanisms may involve phosphorylation-induced protein conformational changes, shifts in cellular redox state, and accumulated cysteine-proximal mutations in malignant cells.^31,33^ Molecular dynamics (MD) simulations^67,68^ have demonstrated that the nucleophilicity of cysteines (and other residues such as lysines) can be conformation-dependent, with kinases providing clear examples of how reactivity varies between functionally relevant conformational states (e.g., DFG-in vs. DFG-out forms). We envision that the contextual information may be added to the training data and at inference stage to provide proteoform-specific predictions in the future. This effort will benefit from the rapid advances in human proteoform discoveries.^69^ Another future development is to enhance LigBind models by including protein-protein interaction (PPI) and cryptic pocket data in training.

AiPP’s demonstrated ability to accurately predict ligandable cysteines and pockets in traditionally undruggable targets such as TFs and PTPs, which not only corroborates but also extend beyond ABPP, supports its broad applicability. Encouraged by results from the extensive application studies, we applied AiPP to create a cysteine-directed ligandability atlas for the human proteome, identifying ligandable sites undetected or unliganded by ABPP experiments. One such example is a predicted allosteric pocket in MC3R, a new target for treatment of eating disorder and obesity. Therefore, we anticipate AiPP will guide future development of chemical probes and pharmaceutical modulators. Finally, the evolutionary-based approach to interrogate, harmonize, and augment experimental labels are broadly applicable to protein research and development of proteomics-based machine learning for diverse applications.

## Supplementary Material

Supplemental Materials

Supplemental Methods contains detailed procedures for constructing the LigCysABPP, LC3D, and LigBind3D databases; pLLM-based data clustering, label assignment and reconciliation; LigCys and LigBind model architectures, training, and validation; and LigCys training data expansion. Supplemental Figures contains a more detailed analysis accompanying Figure 1; model performance analysis using the ABPP hold-out set; and more detailed analysis accompanying Figure 7.

Supplemental Data

Supplemental Data Excel file 1 contains the following 21 sheets: Table of content (SD1). Model benchmark studies: ESMC representation (SD2), consensus (SD3), source ablation (SD4), source prioritization (SD5), option-ablation (SD20) and split-level noise (SD21). Data extraction logs: ABPP extraction log (SD6), log of ambiguous and multivalue entry splitting (SD7), ABPP statistical report (SD8), ABPP orphaned records (dropped due to lack of unambiguous support) (SD9). Sequence and clustering records: validated UID sequences in FASTA format (SD10), UID deduplication log (SD11), precluster records (SD12), cysteine (Cys) clusters report (SD13). Production distillation: 4S–4R distillation report (SD14). Support reports: UID-level (SD15), ROI-level (SD16), and representative-level (SD17). ABPP hold-out set details: UID-level (SD18) and ROI-level (SD19). Optional ablation study (SD20). Split noise study (SD21).

Supplemental Data Excel file 2 contains the data accompanying use case studies: consistently, and heterogeneous cysteines; transcription factors; phosphatases.

Supplementary Files

This is a list of supplementary files associated with this preprint. Click to download.

• AiPPSISept21.pdf

• AiPPSIdata1.xlsx

• SIData2.xlsx

## Figures and Tables

**Table 1:** External evaluation of LigCys models trained using ABPP datasets with varying consensus thresholds^a^

ABPP training data				External LC3D evaluation (%)					
Criteria	Pos	Neg	Prot	PRC	ROC	Prec	Rec	Top-1	Top-1*
	
1S–1R	13430	55441	7051	60.0	72.2	38.4	37.8	40.5	44.5
1S–2R	6078	16791	4216	70.2	80.9	55.9	55.1	56.8	59.6
1S–3R	3396	8600	2829	76.6	85.8	62.3	61.9	65.3	68.2
1S–4R	2094	5048	2004	78.1	86.3	65.3	64.9	68.3	72.9
1S–5R	1418	3354	1495	80.1	87.4	70.0	69.6	71.7	75.9

2S–2R	3467	6838	2617	77.0	84.2	65.3	64.7	66.2	70.3
2S–3R	2198	4765	1883	77.3	85.5	64.3	64.1	66.6	70.3
2S–4R	1450	3326	1404	78.8	86.4	69.6	68.8	70.9	75.1
2S–5R	988	2258	1041	79.5	86.6	71.7	70.9	72.2	76.8

3S–3R	1115	1920	997	78.8	86.6	68.8	68.1	70.0	74.2
3S–4R	867	1583	837	80.2	88.3	68.3	67.5	71.3	75.5
3S–5R	649	1244	669	80.8	87.7	72.6	71.7	73.5	77.6

**4S–4R**	**384**	**642**	**389**	**81.2**	**87.3**	**74.3**	**73.2**	**75.2**	**79.4**
4S–5R	347	590	358	80.3	86.1	74.3	73.2	74.3	78.9

5S–5R	142	236	150	79.8	85.6	71.3	70.3	72.6	76.8

a At a consensus threshold *n*S–*m*R, the label assignment requires at least *m* records from *n* different sources (see main text). Each model is an ensemble of 200 models (see Supplemental Methods). Performance is measured on the orthogonal LC3D testset on the per-protein basis. Cysteines without covalent ligation are provisionally labeled neg. Precision and recall of predicting ligandable cysteines are calculated with a classification threshold of 0.5. Top-1 recovery refers to the probability that the cysteine with the highest classification score is a true pos given that the protein has at least one pos cysteine. Top-1* refers to the evaluation using LC3D augmented with labels from representation-based clustering (see Supplemental data).

**Table 2:** Performance metrics for iterative training data expansion guided by LC3D-based evaluation (top block) and ABPP-based cross validation (bottom block).

ABPP training data^a^				ABPP valid. (%)^b^		External LC3D evaluation^c^ (%)					

LC3D-guided expansion											
Iter	Pos	Neg	Prot	PRCE	ROC	PRC	ROC	Prec	Rec	Top-1	Top-1*

0	354	574	366	74	74.6	77.8	84.9	70.3	69.6	70.9	75.1
0-SA	354	574	366	79	76.5	80.1	86.9	71.3	70.5	72.6	76.8
1	359	649	442	92	78.2	79.4	85.6	71.3	70.3	72.2	76.8
2	369	739	534	93	75.9	81.1	87.5	74.3	73.2	74.4	79.4
3	384	874	674	91	74.2	82.8	88.6	73.9	72.8	76.1	81.1
4	401	1032	827	116	77.5	83.1	88.3	77.7	76.5	77.8	82.4
5	425	1186	966	139	79.3	82.8	87.8	76.9	75.6	77.8	82.0
**6**	**441**	**1345**	**1099**	**141**	**78.2**	**84.2**	**88.6**	**79.0**	**77.7**	**79.9**	**84.5**
6-SA	441	1345	1099	128	76.7	83.8	88.7	76.9	75.6	78.2	83.2

ABPP-guided expansion											
Tour^d^	Pos	Neg	Prot	PRCE	ROC	PRC	ROC	Prec	Rec	Top-1	Top-1*

0	354	574	366	74	74.6	77.8	84.9	70.3	69.6	70.9	75.1
1	378	643	452	93	78.8	80.1	86.8	72.6	71.7	73.1	77.3
2	468	967	817	122	82.1	81.5	88.3	78.1	72.1	74.4	79.4
3	555	1432	1258	147	82.5	80.6	87.8	71.7	70.7	72.6	76.8
**4**	**645**	**1998**	**1744**	**187**	**83.5**	**79.8**	**87.7**	**70.0**	**69.2**	**70.9**	**76.4**
4-SA	645	1998	1744	178	80.9	80.5	87.7	69.8	69.2	71.7	76.8

**Blended** ^e^	-	-	-	-	-	83.4	88.8	76.0	75.0	76.9	82.0

a In the LC3D-guided data expansion, Top-1 recovery of LC3D liganded cysteines was used to select candidate data batches (complete data in Supplemental Data). The total number of pos, neg cysteines, and distinct proteins in the training set is given after each iteration. The start of the expansion (Iter 0) used the 4S–4R^*t*^ dataset, which excludes 23 proteins quantified by 10 sources from the original 4S–4R dataset.

b Model validation metrics are AUROC (ROC) and AUPRC enrichment (PRCE), defined as (AUPRC – random)/random. Here random represents the AUPRC of a random classifier (% positive samples in the dataset).

c For external evaluation against the LC3D dataset, precision (Prec) and recall (Rec) were calculated using the default classification threshold of 0.5. Top-1 and Top-1* were calculated using lC3D and LC3D* (label augmented by clustering) datasets, respectively.

d In the ABPP-guided data expansion, AUPRC enrichment (PRCE) was used to select candidate data batches. Tour denotes the number of successive tournaments (effectively iterations 1, 5, 9, and 13; see Supplemental Data). All other details follow the notes above.

e Blended denotes ensemble prediction using both Iter 6 and Tour 4 models.

**Table 3:** Performance of the sequence-only and structure-aware LigBind models in predicting reversible ligand-binding residues^a^

Metrics (%)	LigBind-seq	LigBind-SA

**AUROC** *	93.9	95.1
**AUPRC** *	74.5	77.5
**Prec** *	72.1	71.1
**Rec** *	66.4	71.5
**Top-5** *	79.2	81.2
**Top-10** *	78.1	80.1
**Top-20** *	76.6	79.5

a All models were trained and evaluated* using cluster-propagated labels derived via representation-based clustering. Performance metrics reflect a post hoc ensemble of 200 models (Supplemental Methods). Per-residue ligandability probabilities were averaged across all 200 models and thresholded at 0.5. All metrics were computed on a per-protein basis using the held-out test set of 129 proteins.

## Data Availability

LigCysABPP and LigBind3D databases and models are available at https://github.com/JanaShenLab/AiPP/. The supplemental data files are also mirrored in the repository. A searchable web server (https://aipp.computchem.org/) provides access to the AiPP atlas of cysteine-directed covalent ligandability in the human proteome, along with ABPP records in the LigCysABPP database.
